# Variability in the Management of Superficial Venous Thrombophlebitis across Practitioners Based in North America and the Global Community

**DOI:** 10.1155/2014/306018

**Published:** 2014-10-12

**Authors:** Anahita Dua, Jennifer A. Heller, Bhavin Patel, Sapan S. Desai

**Affiliations:** ^1^Department of Surgery, Medical College of Wisconsin, 8701 Watertown Plank, Milwaukee, WI 53045, USA; ^2^Division of Vascular Surgery, Department of Surgery, Johns Hopkins Medical Center, Baltimore, MD, USA; ^3^Department of Internal Medicine, North Shore-Long Island Jewish Health System, New York City, NY, USA

## Abstract

*Introduction*. This study aimed to compare management patterns of patients with SVT among healthcare practitioners based in North America versus those in the global community.* Methods*. A 17-question, multiple choice survey with questions regarding SVT diagnosis and management strategies was provided to practitioners who attended the American Venous Forum (AVF) meeting in 2011.* Results*. There were 487 practitioners surveyed with 365 classified as North American (US or Canada) and 122 (56 Europe, 25 Asia, 11 South America, and 7 Africa) representing the global community. The key difference seen between the groups was in the initial imaging study used in patients presenting with SVT (*P* = 0.046) and physicians in the US ordered fewer bilateral duplex ultrasounds and more unilateral duplex ultrasounds (49.6% versus 58.2%, 39.7% versus 34.4%). In the US cohort, phlebologists and vascular surgeons constituted 82% (*n* = 300) of the specialties surveyed. In the global community, SVT was managed by phlebologists or vascular surgeons 44% (*n* = 54) of the time. Surgical management was highly variable between groups.* Conclusion*. There is currently no consensus between or among practitioners in North America or globally as to the surgical management of SVT, duration of follow-up, and anticoagulation parameters.

## 1. Introduction

The diagnosis and management of superficial venous thrombophlebitis (SVT) is poorly defined and remains controversial both within practitioners here in the USA and globally [1]. SVT is a relatively common disease with up to an 11% incidence rate [1–3]. While SVT used to be considered a self-limiting disease, benign disease studies have confirmed the close correlation between SVT and deep-vein thrombosis (DVT) or pulmonary embolism (PE) [1–4]. DVT or PE is diagnosed in up to 20–30% of patients with SVT with clinically relevant symptomatic thromboembolic events complicating isolated SVT in 4–8% of patients [3, 4].

SVT is managed by a variety of practitioners including family practice physicians, dermatologists, internists, phlebologists, cardiologists, interventional radiologists, general surgeons, and vascular surgeons resulting in further variations in treatment regimes. Different countries have adopted variable practice patterns for this disease and at present no consensus had been reached regarding optimum care. A 2013 Cochrane review attempting to delineate most favorable treatment patterns for SVT concluded that while prophylactic doses of fondaparinux given for six weeks appear to be a valid therapeutic option for SVT of the legs, the current published evidence on oral treatments, topical treatment, or surgical intervention was too limited to make any conclusions [1]. Nationally and internationally the treatment of SVT ranges from medical management inclusive of compression stockings, limb elevation, pain control, and low molecular weight heparin (LMWH) to surgical intervention such as sclerotherapy, high ligation, vein stripping, phlebectomy, or thermal ablation. Timing and type of therapy are healthcare provider and location dependent, therefore varying substantially. While the global gold standard SVT management consensus involves attempts at medical management followed by surgical intervention in those patients who present with “extensive disease” or “recurrence,” what constitutes medical management, recurrence, or extensive disease is not defined [1–4]. To our knowledge, no research has been conducted reviewing the practice pattern differences between healthcare providers in North America compared to the rest of the world for the management of SVT.

This study aims to compare practice patterns of practitioners based in North America to those based globally in regard to the diagnosis and management of patients with SVT.

## 2. Methods

A survey with questions regarding SVT diagnosis and management strategies was provided to all practitioners who attended the American Venous Forum (AVF) meeting in 2011. Survey instructions stated that participating practitioners should include patients who had thrombosis of the superficial veins of the extremities (saphenous vein, superficial tributaries, and varicose veins), with or without symptoms, but without extension into the deep vein system.

### 2.1. Question Development and Survey Generation

We developed a questionnaire using the previous literature. The questionnaire consisted of seventeen closed-ended questions in multiple-choice format. The following is a list of the questions that comprised the survey.

1. If a patient presents with symptoms and physical findings suspicious for SVT of a lower extremity, what kind of duplex ultrasound do you order?
2. After diagnosing SAPHENOUS vein thrombophlebitis, when do you repeat a duplex ultrasound study?
3. After diagnosing SVT involving only superficial tributaries and/or varicosities (NOT the saphenous vein), when do you repeat a duplex ultrasound study?
4. When do you order blood testing for thrombophilia in patients with SVT?
5. When do you check cancer screenings in patients with SVT?
6. How do you distinguish acute from chronic SVT?
7. When do you anticoagulate (e.g. heparin, low molecular weight heparins, warfarin) patients with ACUTE GSV thrombophlebitis?
8. When do you recommend intervention (e.g. ligation, stripping, thermal ablation, chemical ablation) for ACUTE GSV thrombophlebitis?
9. When do you anticoagulate (e.g. heparin, low molecular weight heparins, warfarin) patients with ACUTE SSV thrombophlebitis?
10. When do you recommend intervention (e.g. ligation, stripping, thermal ablation, chemical ablation) for ACUTE SSV thrombophlebitis?
11. If you choose to anticoagulate a patient for their first episode of acute SVT, what is the duration of treatment?
12. Please rank in order your preference for each procedure for CHRONIC GSV thrombophlebitis, assuming you feel the procedure is both indicated and technically feasible?
13. When do you follow up with a patient with ACUTE SAPHENOUS thrombophlebitis?
14. When do you follow up with a patient with ACUTE SVT involving only superficial tributaries and/or varicosities (NOT the saphenous vein)?
15. How do you recommend initially treating patients with superficial thrombophlebitis in GSV tributaries after technically successful GSV ablation?
16. How do you recommend initially treating patients with trapped blood in varicosities after sclerotherapy?
17. Please indicate your specialty.

### 2.2. Questionnaire Administration

All active members of American Venous Forum (AVF) 2011 were surveyed voluntarily. Potential participants were given the survey with no monetary incentives. All response data was collected anonymously and grouped according to predefined analyses. Individual responses were kept confidential and questionnaire completion was voluntary.

### 2.3. Statistical Analysis

Statistical analysis was performed with descriptive statistics and paired *t*-tests with *P* < 0.05 deemed statistically significant.

## 3. Results

There were 487 practitioners surveyed with 365 classified as North American (US or Canada) and 122 (56 Europe, 25 Asia, 11 South America, and 7 Africa) representing the global community. There was wide variation in the specialties that were primarily responsible for the management of SVT in North America versus the global community.

Table 1 depicts the breakdown in specialty of the practitioners tasked with treating patients with SVT.

Data was divided and analyzed comparatively in four groups: initial follow-up imaging, preferred surgical intervention (high ligation, high ligation and stripping, thermal ablation, chemical ablation, and none), anticoagulation trends, and follow-up trends between the North American cohort and the global community.

Table 2 depicts the initial and follow-up imaging studies for diagnosing SVT among North American practitioners and the global community.

There was a significant difference in the type of initial duplex performed (*P* = 0.046) but no difference when North American based or global community based practitioners ordered repeat scans after initial diagnosis.

Table 3 depicts preferred surgical intervention comparing North American practitioners and the global community.

Friedman's statistical analysis on these five surgical interventions resulted in a nonsignificant *P* value. This indicated that, of the five treatments, high ligation, high ligation and stripping, thermal ablation, chemical ablation, and no treatment, none were ranked higher than the others between North American practitioners or practitioners based in the global community.

Table 4 depicts anticoagulation trends among North American practitioners or practitioners based in the global community.

In our data set, there were no differences noted when anticoagulation was initiated or in the duration of the treatment between groups.

Table 5 depicts follow-up trends among North American practitioners or practitioners based in the global community after treating acute SVT.

North American practitioners and practitioners within the global community differed in the time to follow-up imaging for patients with acute SVT (*P* < 0.005) and how they treated patients after successfully ablating the greater saphenous vein or in patients with trapped blood after sclerotherapy (medical management, compression therapy) (*P* < 0.005).

## 4. Discussion

Our research aimed to report on the differences in practice patterns amongst practitioners in North America as compared to the rest of the world in the diagnosis and management of patients with SVT. To our knowledge, this is the only research conducted on this topic.

SVT has long been considered a benign disease with minimal complications; recent studies have shown that lethal complications arising from VTE may be higher than previously expected [5–7]. Our study found there are significant intercountry and interspecialty variations in the management of patients with SVT. Given the disease burden of SVT and possibility of more dire consequences (VTE) it is important to develop a clear consensus for optimum management of these patients.

Our study compared surgical interventions utilized in the management of SVT in North America versus the global community. A key difference seen between the groups was in the initial imaging study used in patients presenting with SVT, as North American practitioners ordered fewer bilateral duplex ultrasounds and more unilateral duplex ultrasounds compared to their global colleagues. Interestingly, within management options, we found that all forms of surgical intervention including high ligation, ligation with stripping, chemical ablation, or thermal ablation were performed amongst practitioners without any preference for one over another. This data suggests that there is no consensus regarding principles that guide interventional management in patients with SVT. Differences also persisted in posttreatment management and follow-up times with North American practitioners much more likely to order follow-up imaging within the first week posttreatment.

The reason for variations in management may be based on the fact that different healthcare practitioners treat SVT in both North America and the globe. In the US cohort, phlebologists and vascular surgeons constituted 82% (*n* = 300) of the specialties surveyed. In the global community, SVT was managed by phlebologists or vascular surgeons only 44% (*n* = 54) of the time; other top specialties who primarily managed SVT included 13% (*n* = 13) dermatology, 7.4% (*n* = 9) interventional radiology, 7.4% (*n* = 9) hematology, or 7% (*n* = 8) cardiology. This difference in the type of practitioner that manages SVT may be the reason for the differences noted within groups in our study. Lozano and Almazan [8], a group of vascular surgeons based in Spain, reported in their prospective study that the preferred surgical treatment in a prospective study was a high ligation at the saphenofemoral junction [8]. However, Belcaro et al. [9] from Italy compared the efficacy of five different therapeutic methods including compression, surgery, low-dose subcutaneous heparin, LMWH, and oral anticoagulation and failed to conclude which treatment was the best practice [9]. From North America, Sullivan et al. [10] reported in 2001 that few differences existed in their study between medically managed and surgically managed patients and also could not formally conclude which was the best option [10].

While many studies have established variations in surgical treatment based specialty, few studies have attempted to define variations among countries and/or specialties especially for a disease such as SVT process that is routinely managed by over ten different types of practitioners worldwide. [11, 12] As a result, variations may arise from insufficient knowledge of or disagreement with guidelines among physicians, inadequate communication between physicians and patients, and individual preferences or clinical attributes of patients [13].

The inconsistencies among practitioners in part may be due to the fact that SVT was managed by a much more diverse group of practitioners in terms of their specialties globally. Our data shows that not only SVT is managed differently depending on the location, but SVT is managed differently among practitioners within the same region.

*Limitations*. Due to the survey based nature of this study, it was not possible to define some of the granular details of treatment associated with the management practices of these practitioners. Specifically, we were unable to ascertain how chronic luminal changes were managed and how recurrent episodes were treated as this study focused primarily on acute, first time episodes of SVT.

## 5. Conclusion

There are differences in SVT management strategies between practitioners managing SVT in North America versus the global community. These differences may be attributed to the fact that a variety of other specialties aside from phlebology and vascular surgery are involved in the care of these patients. Our data suggests that there is currently no consensus between or amongst practitioners in North America or globally as to the surgical management of SVT, duration of follow-up, and anticoagulation parameters. Further studies reviewing patient outcomes are warranted to delimitate the optimum course of management of SVT.

## Figures and Tables

**Table 1:** Breakdown in specialty of the practitioners tasked with treating patients with SVT.

Variable	Global community (*n*)	Global community (%)	North America (*n*)	North America (%)
Phlebology	26	21.31	143	39.13
Vascular surgery	28	22.95	157	43.01
General surgery	5	4.1	10	2.74
Interventional radiology	9	7.38	10	2.74
Dermatology	16	13.11	10	2.74
Hematology	9	7.38	9	2.47
Cardiology	8	6.56	10	2.74
Other	14	11.48	9	2.47
No answer	7	5.74	7	1.92
Total	**122**	**100**	**365**	**100**

**Table 2:** Initial and follow-up imaging studies for diagnosing SVT among practitioners in North America and the global community.

Variable	North America, *n* (%)	Global Community, *n* (%)	*P* value
Type of initial duplex ultrasound			0.046
Bilateral lower extremity	181 (49.6%)	71 (58.2%)	
Unilateral lower extremity	145 (39.7%)	42 (34.4%)	
No ultrasound needed	22 (6%)	7 (5.7%)	
No answer	17 (4.7%)	2 (1.6%)	

After diagnosis of saphenous SVT, repeat ultrasound			0.88
1 week or less	105 (28.8%)	39 (32%)	
1–4 weeks	63 (17.3%)	23 (18.9%)	
1–3 months	52 (14.3%)	11 (9%)	
Only if symptoms worsen	62 (17%)	17 (13.9%)	
Other/no answer	83 (22.7%)	32 (26.2%)	

After diagnosis of SVT of superficial tributaries, repeat ultrasound			0.30
1 week or less	74 (20.3%)	28 (23%)	
1–4 weeks	64 (17.5%)	23 (18.9%)	
1–3 months	50 (13.7%)	19 (15.6%)	
Only if symptoms worsen	95 (26%)	28 (23%)	
Other/no answer	82 (22.5%)	24 (19.7%)	

**Table 3:** Surgical intervention preference, North America versus global community.

	High ligation	High ligation with stripping	Thermal ablation	Chemical ablation	None
North America	21.64%	16.99%	22.47%	19.45%	18.90%
Global community	20.49%	21.31%	19.67%	21.31%	18.03%

**Table 4:** Anticoagulation trends among practitioners in North America and the global community.

Variable	North America, *n* (%)	Global community, *n* (%)	*P* value
Anticoagulation for patients with acute GSV SVT			0.84
All patients	34 (9.3%)	12 (9.8%)	
Involvement of >5 cm GSV	37 (10.1%)	16 (13.1%)	
Clot within 10 cm of saphenofemoral junction	101 (27.7%)	34 (27.9%)	
Proximal extension of clot on follow-up visit	91 (24.9%)	25 (20.5%)	
Never	41 (11.2%)	15 (12.3%)	
Other/no answer	61 (16.7%)	20 (16.4%)	

Anticoagulation for patients with acute SSV SVT			0.10
All patients	48 (13.2%)	11 (9%)	
Involvement of >5 cm SSV	44 (12%)	13 (10.7%)	
Clot with 10 cm of saphenofemoral junction	68 (18.6%)	25 (20.5%)	
Proximal extension of clot on follow-up visit	75 (20.6%)	38 (31.2%)	
Never	50 (13.7%)	11 (9%)	
Other/no answer	80 (21.9%)	24 (19.7%)	

Duration of initial anticoagulation for acute SVT			0.14
1 month or less	69 (18.9%)	28 (23%)	
1–3 months	95 (26%)	38 (31.2%)	
4–6 months	60 (16.4%)	17 (13.9%)	
>6 months	44 (12.1%)	13 (10.7%)	
Other/no answer	97 (26.6%)	26 (22.1%)	

**Table 5:** Follow-up trends among practitioners in North America and the global community.

Variable	North America, *n* (%)	Global community, *n* (%)	*P* value
Follow-up of patients with acute saphenous thrombophlebitis			<0.005
<1 week	142 (38.9%)	21 (17.2%)	
1–4 weeks	102 (28.0%)	24 (19.7%)	
1–3 months	30 (8.2%)	15 (12.3%)	
Only if symptoms worsen	25 (6.9%)	25 (20.5%)	
Other/no answer	66 (18.1%)	37 (30.3%)	

Follow-up for patients with SVT of superficial tributaries/varicosities besides saphenous vein			0.27
<1 week	72 (19.7%)	26 (21.3%)	
1–4 weeks	64 (17.5%)	20 (16.4%)	
1–3 months	51 (14.0%)	21 (17.2%)	
Only if symptoms worsen	54 (14.8%)	22 (18.0%)	
Other/no answer	124 (34.0%)	33 (27.1%)	

Treating patients with SVT after successful GSV ablation			<0.005
Compression and NSAIDs	225 (61.6%)	39 (32.0%)	
Immediate clot drainage	98 (26.9%)	36 (29.5%)	
Other/no answer	42 (11.5%)	47 (38.5%)	

Initially treating patients with trapped blood after sclerotherapy			<0.005
Compression and NSAIDs	123 (33.7%)	37 (30.3%)	
Immediate clot drainage	198 (54.3%)	42 (34.4%)	
Other/no answer	43 (12.1%)	43 (35.3%)	
